# Enhanced Thermal Conductivity of Thermoplastic Polyimide Nanocomposites: Effect of Using Hexagonal Nanoparticles

**DOI:** 10.3390/polym16233231

**Published:** 2024-11-21

**Authors:** Victor M. Nazarychev

**Affiliations:** Branch of Petersburg Nuclear Physics Institute Named by B.P. Konstantinov of National Research Centre «Kurchatov Institute»—Institute of Macromolecular Compounds, Bolshoi, pr. 31 (V.O.), 199004 St. Petersburg, Russia; nazarychev@imc.macro.ru; Tel.: +7-(812)-3230216

**Keywords:** thermal interface materials, polyimides, thermal conductivity, molecular dynamics, nanocomposite, graphene, graphene oxide, hexagonal boron nitride

## Abstract

Thermoplastic polyimides have garnered significant interest in the electronic and electrical industries owing to their performance characteristics. However, their relatively low thermal conductivity coefficients pose a challenge. To address this issue, this study focused on the properties of nanocomposites comprising two thermoplastic semicrystalline polyimides R-BAPB and BPDA-P3, one amorphous polyimide ULTEM^TM^, and hexagonal nanoparticles. Polyimide R-BAPB was synthesized based on 1,3-bis-(3′,4-dicarboxyphenoxy)benzene (dianhydride R) and 4,4′-bis-(4′-aminophenoxy)biphenyl (BAPB diamine); polyimide BPDA-P3 was synthesized based on 3,3′,4,4′-biphenyltetracarboxylic dianhydride (BPDA) and diamine 1,4-bis[4-(4-aminophenoxy)phenoxy]benzene (P3); and amorphous polyimide ULTEM^TM^ was commercially produced by Sabic Innovative Plastics. Using microsecond-scale all-atom molecular dynamics simulations, the effects of incorporating hexagonal nanoparticles with enhanced thermal conductivity, such as graphene, graphene oxide, and boron nitride, on the structural and thermophysical characteristics of these materials were examined. The formation of stacked aggregates was found for graphene and hexagonal boron nitride nanoparticles. It was observed that graphene oxide nanoparticles exhibited a dispersion in polyimide binders that was higher than those in graphene and hexagonal boron nitride nanoparticles, leading to reduced translational mobility of polymer chains. Consequently, the decrease in polyimide chain mobility correlated with an increase in the glass transition temperature of the nanocomposites. Aggregates of nanoparticles formed a pathway for phonon transport, resulting in improved thermal conductivity in polyimide nanocomposites. An increase in the thermal conductivity coefficient of polyimide nanocomposites was observed when the concentration of graphene, graphene oxide, and hexagonal boron nitride nanofillers increased. The enhancement in thermal conductivity was found to be strongest when graphene nanoparticles were added.

## 1. Introduction

The thermal conductivity of materials is essential in engineering applications, especially in the advancement of thermal interface materials [1]. Thermal interface materials can efficiently dissipate heat from heated surfaces [2]. This can significantly improve the performance, durability, and cost required to maintain the operation [3]. Polymers are promising materials for the fabrication of thermal interface materials due to their strength, light weight, low cost, specific surface–weight ratio, and good insulating properties [4]. However, the thermal conductivity of polymers is not sufficiently high, presently not exceeding 0.5 W/m·K [5].

Thermoplastic polyimides (PIs) are polymers that exhibit excellent dielectric and mechanical characteristics as well as strong resistance to radiation and chemical attacks [6], which have resulted in them attracting substantial interest. Unfortunately, the thermal conductivity characteristics of these polymers remain low because they are similar to those of other polymers, restricting their use in fields that require effective heat dissipation, such as flexible electronics and thermal management materials [4]. The thermal conductivity of polymers can be improved by adding nanoparticles with excellent heat-conducting properties to the polymer binder [7]. Metal oxide (MeO) nanoparticles are considered one of the most effective materials for heat transmission due to their high thermal conductivity and relatively high resistance to heat [8]. While MeO nanoparticles have a low dispersion ability in polymers, these nanoparticles can be modified on the surface to make them compatible with polymer binders [9].

Graphene and graphene oxide are the carbon nanoparticles with the highest thermal conductivity [10] and elevated electrical conductivity, potentially influencing the dielectric characteristics of the polymers. To improve the dispersion of nanoparticles within the polymer binder [11], graphene oxide nanoparticles with hydroxyl and epoxy groups on their surfaces are often used instead of graphene. However, this modification of the graphene surface results in a lower thermal conductivity of the graphene oxide than that of graphene [12]. Hexagonal boron nitride nanoparticles [10,13,14,15] may be substitute for graphene and graphene oxide nanoparticles. Although they have a hexagonal cellular structure similar to that of graphene, they lack electrical conductivity and exhibit high thermal conductivity [16]. Owing to these properties, it is advisable to improve the ability of PI compounds to control heat [14,17,18]. Extended experimental studies related to the investigation of the thermal conductivity and heat resistance of thermal interface materials were conducted by Prof. Balandin’s group [19,20,21]. They investigated the influence of the thickness and lateral dimensions of graphene and boron nitride nanoparticles on the electrical conductivity [19], phonon thermal transport in noncured silicone composites [20], and thermal conductivity properties of graphene-based composites at cryogenic temperatures [21]. It can therefore be seen that the embedding of nanomaterials such as graphene, graphene oxide, and hexagonal boron nitride into polymer matrices has shown promising results in enhancing thermal conductivity.

Molecular dynamics (MD) simulations are an effective method for studying atomic-level heat-transport processes and interfacial interactions in nanocomposite systems [22,23]. MD allows for the examination of the impact of varying the chemical structures of the nanofiller and polymer on the thermal conductivity of polymer materials [24,25]. Prior work in this area can be divided into three main categories based on the type of nanofiller: graphene, graphene oxide, and hexagonal boron nitride. Several studies [26,27,28,29,30] have investigated the effect of graphene nanoparticles on the thermophysical properties of polymer nanocomposite materials. The various mechanisms that lead to the enhancement of thermophysical properties of composites materials have been considered. Chen et al. [26,27] used MD simulations to ascertain the presence of a physical mechanism that causes thermal percolation in a composite system consisting of graphene and a polymer. The heat conductivity of the polymer nanocomposites increased by over 150% when covalent connections were formed between the graphene sheets. The authors showed [27] that the thermal conductivity of polymers is improved by adding graphene nanoplatelets and that the orthogonal arrangement of graphene exhibits higher thermal conductivity in polymer composites. Yang et al. [28] showed that graphene effectively improves the thermal conductivity of different polymer matrices (polyamide 6, polypropylene, and high-density polyethylene) and demonstrated the strong impact of graphene on the thermal conductivity of polymer nanocomposites. The influence of defective graphene and graphene oxide on the thermal conductivity and mechanical properties of nanocomposites has been shown [29]. The thermal conductivities of polymer nanocomposites with both nanoparticles exhibited a positive correlation with the weight fraction, with a notable increase observed when the weight fraction exceeded 20 wt%. Luo et al. [30] focused on a systematic investigation of thermal energy transfer in composite systems consisting of graphene/graphite and a polymer. Enhancement of the interfacial thermal transport on the basis of the simulation findings can be achieved by increasing the polymer density and the graphene size or by creating a covalent bond between the graphene edges and polymer chains. However, the flat hexagonal structure of the graphene surface might lead to possible aggregation of the graphene nanosheets in the polymer samples.

The change from graphene to graphene-like materials, such as graphene oxides and hexagonal boron nitride, could facilitate the dispersion of nanoparticles in the polymer matrix to a considerable degree. Rahmani et al. [31] investigated the impact of the concentration of graphene and graphene oxide on the thermal stability of poly(ethylene oxide) and showed that the thermal stability of the polymer nanocomposites increased with the increasing mass fraction of graphene. The strong correlation between the slowing dynamics of polymer chains at the interface and their interactions with the nanofillers was found [32]. At the same time, the thermal transport of phonons upon the embedding of graphene oxides in the polymers demonstrated the influence of the number of oxygen groups on the thermal conductivity of reduced graphene oxide [33]. Bellussi et al. [34] found that the degree of oxygenation of the graphene filler has a significant impact on the interaction with polar polymers, which decreased Kapitza resistance and improved thermal conductivity at the interface. Present modification of the graphene surface by grafting hydroxyl or epoxy groups to its surface should have a better effect on heat transport at the ‘polymer–nanofiller’ interface [35,36]. The researchers discovered that the presence of hydroxyl groups and vacancy defects might increase the thermal conductivity in graphene oxide films [35] and the strong influence of the concentration of oxygen groups in graphene oxide on mechanical and thermophysical properties [36].

Evidence from other simulation studies [37,38,39] suggests that the similar hexagonal chemical structure of boron nitride nanoparticles could contribute to a significant increase in the value of thermal conductivity coefficient. For instance, Zhu et al. [37] studied the influence of the form (nanoparticles, nanotubes, and nanosheets), volume fractions, and aspect ratios of boron nitride nanofillers on the performance characteristics of epoxy resin nanocomposites, improving their thermal conductivity and mechanical properties. An increase in the percentage of nanofiller mass and oscillatory shear was found to affect heat transfer in a nanocomposite in-plane direction composed of poly(dimethylsiloxane) (PDMS) and hexagonal boron nitride (h-BN) nanoparticles [38]. Additionally, Zhang et al. [39] showed that the density and distribution of defects in h-BN nanoparticles have an impact on the thermal conductivity of PDMS/h-BN nanocomposites, leading to an increase in the interfacial thermal conductivity while reducing the intrinsic thermal conductivity, which affects the total thermal conductivity of the nanocomposites. However, the systematic influence of the mass fraction of three types of nanoparticles (graphene, graphene oxide, and hexagonal boron nitride) on the thermophysical, structural, and dynamic properties of PI nanocomposites has not been previously investigated using atomistic molecular dynamics.

**Figure 1:**
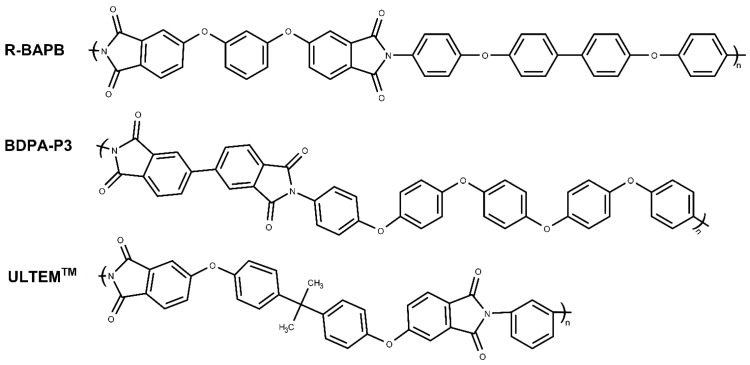
Chemical structures of the repeating units of the thermoplastic polyimides R-BAPB, BPDA-P3, and ULTEM^TM^.

In this study, the impact of graphene, graphene oxide, and hexagonal boron nitride nanoparticles on the thermal conductivity of PIs was analyzed using molecular dynamics simulations. The effect of the mass fraction on the thermal conductivity of nanocomposites based on these nanoparticles was examined. A thermoplastic PI R-BAPB was selected as the object of this study. This semicrystalline PI R-BAPB was synthesized and developed in Institute of Macromolecular Compounds RAS [40,41]. The structural, thermal, and mechanical properties of PIs have been studied extensively [42,43,44,45,46]. Furthermore, in this work, the effect of the concentration of considered nanoparticles on the thermophysical and structural properties of two polyimides, semicrystalline BPDA-P3 [47,48] and amorphous ULTEM^TM^, was investigated. The chemical structures of three PIs are shown in Figure 1.

Previously [49], our study showed that the application of uniaxial deformation could significantly affect the thermal conductivity of both semicrystalline and amorphous PIs. In this study, the influence of hexagonal nanofillers on the thermophysical and structural properties of three PIs will be investigated. By exploring the nanoscale interactions, the effects of incorporation of these nanofillers on the thermal conductivity of the PI nanocomposites will be studied. This research contributes to the fundamental understanding of nanocomposite materials and paves the way for the design of advanced thermal interface materials with improved thermal conductivity for various industrial applications.

## 2. Methodology

### 2.1. Objects of Study

In this study, the structural and thermophysical properties were simulated of polymer nanocomposites based on three thermoplastic polyimides—semicrystalline R-BAPB based on 1,3-bis-(3′,4-dicarboxyphenoxy)benzene (dianhydride R) and 4,4′-bis-(4′-aminophenoxy)biphenyl (BAPB diamine) [50,51]; semicrystalline BPDA-P3 based on 3,3′,4,4′-biphenyltetracarboxylic dianhydride (BPDA) and diamine 1,4-bis[4-(4-aminophenoxy)phenoxy]benzene (P3) [48]; and amorphous polyimide ULTEM^TM^, which is commercially produced by Sabic Innovative Plastics [52] (Figure 1)—by embedding them with graphene, graphene oxide, and hexagonal boron nitride nanoparticles.

### 2.2. Model and Simulation Techniques

Computer simulations were performed using the atomistic molecular dynamics package Gromacs (v. 2022) [53]. As reported in a previous study [49], the polymer chains of the investigated PIs consisted of eight repeating units. Simulations of the considered systems were performed using a GAFF force field [54]. It was previously shown that the GAFF force field allows the reproduction of the ratio of the thermal properties of these polymers in a simulation. In addition, in previous studies [55,56,57], the use of the GAFF force field demonstrated the ability to predict the thermal conductivity of paraffin and paraffin-based nanocomposites. The partial charges were calculated using the HF/6-31G* (RESP) partial charge calculation method.

**Figure 2:**
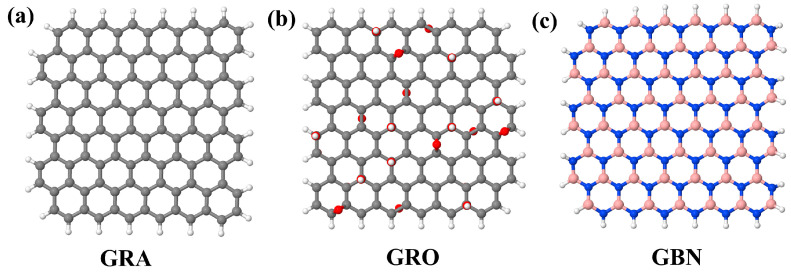
Chemical structure of the molecules of (**a**) graphene (‘GRA’), (**b**) graphene oxide (‘GRO’), and (**c**) hexagonal boron nitride (‘GBN’).

To investigate the influence of nanofiller concentration on the thermal conductivity of PI nanocomposites, three nanofillers (graphene, graphene oxide, and boron nitride) with similar hexagonal chemical structures were chosen, as shown in Figure 2. The number of carbon atoms in the aromatic backbone of graphene and graphene oxide molecules, as well as the total number of nitrogen and boron atoms in the backbone of hexagonal boron nitride, was chosen to be the same and was equal to 138. To build the graphene oxide model, the hydroxyl and epoxy groups were randomly sewn on the surface of graphene; thus, the ratio of carbon and oxygen atoms was close to 5:1, and the ratio of the hydroxyl and epoxy groups was 3:2 [58,59]. Thus, the total number of atoms in the graphene oxide molecule was 183.

The intramolecular and intermolecular interactions of the nanoparticles were described using the GAFF force field [60]. Partial charges were calculated using the R.E.D. program [61] and the Gaussian 09 quantum chemistry package [62]. The ACPYPE package was used to parameterize the graphene and graphene oxide structures using the GAFF force field [63,64]. The parameters for boron nitride were obtained from previous studies [54,65,66,67].

To create the initial configuration for the unfilled PI chains, 27 randomly oriented polymer chains were placed in a simulation box [49]. The initial side length of the simulation box was 50 nm. To create the polymer nanocomposites, 6, 12, 24, or 48 nanofiller molecules were added to the simulation box, and further systems were compressed under a pressure of 50 bar at *T* = 800 K for 20 ns, Table 1. It should be noted that the change in the number of hexagonal nanoparticles was carried out in such a wide range in an attempt to find the trend between the change in the thermophysical and structural properties of the considered systems. In future studies, the electrical conductivity and dielectric properties of the created polyimide nanocomposites will be investigated.

After compression, the simulation was run for 1.0–1.5 μs. This time was sufficient for the size of the polymer chains to reach values in good agreement with the theoretical predictions [68], as shown in Figure S1 in the Supplementary Materials. The V-rescale thermostat [69] and Parrinello–Rahman barostat [70] were used during preliminary equilibration. After system equilibration, the V-rescale thermostat was changed to a Nose–Hoover [71] thermostat. The samples were then cooled from 800 K to 290 K at a cooling rate of 1.5 × 10^11^ K/min, during which the temperature dependence of the density (*ρ*(*T*)) was calculated [72]. *ρ*(*T*) was used to determine the glass transition temperatures (*T_g_*) of the PI nanocomposites. Two distinct linear regions can be identified in the *ρ*(*T*) plot: one at a low temperature and the other at a high temperature. *T_g_* is defined as the temperature at which the two linear regions intersect in the *ρ*(*T*) curve.

The average aggregate size and the average number of aggregates were evaluated to characterize the aggregation ability of the nanoparticles in the polymer binder. The Gromacs procedure (gmx clustsize) was used to evaluate these characteristics. Similarly to previous studies [73,74,75], a cut-off radius of 0.45 nm was chosen, which is the smallest atomic distance between the considered nanoparticles.

Furthermore, to study the structure of the nanocomposites, the radial distribution functions (RDFs) between the nanoparticle center of mass or atoms was calculated:

(1) $$g_{A-B}\left(r\right)=\frac{1}{N_{A}\langle \rho_{B}\rangle }\sum_{i\in A}^{N_{A}}\sum_{j\in B}^{N_{B}}\frac{\delta \left(r_{ij}-r\right)}{4\pi r^{2}},$$

where A and B are the number of atoms in atom type *A* and atoms in atom type *B*, respectively; $\left\langle \rho_{B}\right\rangle$ is the average density of atom type *B* located in a sphere of radius r from atom type *A*; $r_{ij}\;$is the distance between two atoms *i* and *j* related to atom type *A* and atom type *B*; and δ is the Kronecker delta.

The mean squared displacement (MSD) of the center of mass of the polymer chains and nanofiller molecules [42] was calculated as follows:

(2) $$\langle {\vec{r}}^{2}\left(t\right)\rangle =\langle {\left[\vec{r}\left(t_{0}+t\right)-\vec{r}\left(t_{0}\right)\right]}^{2}\rangle ,$$

where $\vec{r}\left(t_{0}\right)$ is the radius-vector of the center of mass of a polymer chain (nanofiller molecule) at time *t*_0_ and $\vec{r}\left(t_{0}+t\right)-\vec{r}\left(t_{0}\right)$ is the distance, which the center of mass of the polymer chain or nanoparticle molecule runs during time interval *t*. The brackets <…> indicate that averaging was performed over all polymer chains (nanoparticle molecules) and times *t*_0_. Mean squared displacements of the centers of mass of individual polymer chains and three types of nanoparticles are shown in Figures S2–S4 in the Supplementary Materials.

The equilibrium molecular dynamics (EMD) method was used to determine the thermal conductivity coefficient (*κ*). The values of *κ* were calculated using the method implemented in LAMMPS package (version from 15 April 2020) [76]. For this purpose, the topology and coordinate files were converged from Gromacs to LAMMPS representation using a previously developed methodology [55]. In the EMD method, the autocorrelation function of the heat flux was integrated [77] using the Green–Kubo equation [78,79]. The correction of virial for many–body interactions such as valence and dihedral angle potentials was implemented [80,81]. To compare the thermal conductivity properties of the considered systems, the average *κ* was calculated as the arithmetic mean of the diagonal components (*κ_xx_*, *κ_yy_*, and *κ_zz_*) of the thermal conductivity tensor. Nevertheless, it should be noted that in general, the thermal conductivity tensor of the investigated systems was anisotropic, and one should be very careful when comparing the thermal conductivity properties of the investigated systems. The molecules of the hexagonal nanoparticles formed stacked aggregates with an inhomogeneous structure in simulation box, leading to anisotropic thermal conductivity. By analogy with the experimental studies, the average values of the diagonal components of the thermal conductivity tensor were calculated to quantitatively compare the influence of the mass fraction of different nanofillers on the thermal conductivity of the nanocomposites.

## 3. Results and Discussion

### 3.1. Structural Properties

Initially, the interactions between graphene, graphene oxide, and boron nitride nanoparticles in polymer nanocomposites were examined. To achieve this, RDFs for the centers of mass of nanoparticles were computed at *T* = 800 K.

**Figure 3:**
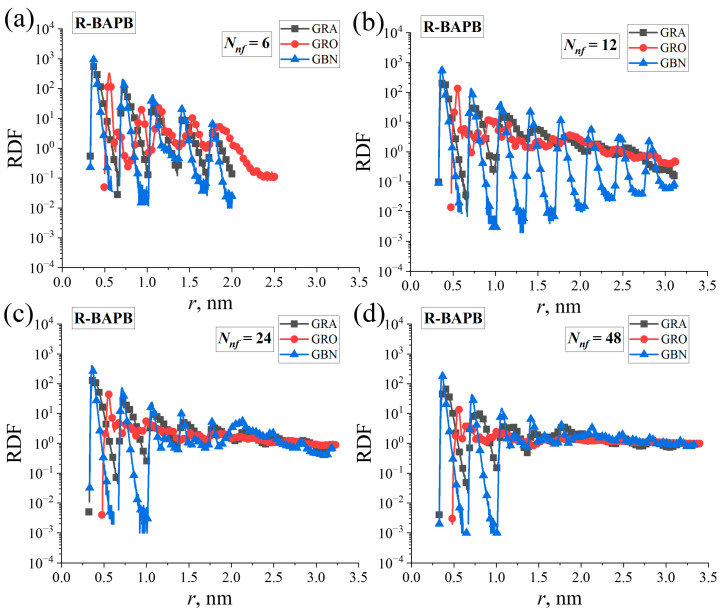
Radial distribution functions (RDFs) for the centers of mass of the polycyclic cores of graphene (‘GRA’), graphene oxide (‘GRO’), and hexagonal boron nitride (‘GBN’) molecules in R-BAPB PI nanocomposite systems for various numbers of *N_nf_*: (**a**) 6, (**b**) 12, (**c**) 24, and (**d**) 48 nanofiller molecules.

Figure 3 displays the RDFs of the centers of mass of nanoparticles in R-BAPB-based nanocomposites. Similar dependencies of the RDFs for center of mass of nanoparticles in BPDA-P3 and ULTEM^TM^ nanocomposites were calculated, as shown in Figures S5 and S6 in the Supplementary Materials.

Changes in the structure of the nanoparticle aggregates in PI nanocomposites with increasing mass fraction of the nanofiller were analyzed. To do this, the RDFs of the center of mass of the polycyclic cores of studied nanoparticles were estimated. Analysis of the data indicated that the RDFs between the centers of mass of graphene and hexagonal boron nitride molecules exhibited a similar pattern. Multiple peaks (ranging from three to nine) were observed on the RDFs, suggesting the presence of long-range order among the nanoparticles within the simulation box. This finding might confirm the formation of more pronounced aggregates for these nanoparticles. The RDFs between the centers of mass of graphene oxide molecules exhibited slight variations: the RDFs displayed multiple peaks, but with a reduced magnitude. Additionally, the position of the first peak shifted to a greater distance owing to the presence of hydroxyl and epoxy groups on the surface of graphene oxide. 

A decrease in the peak height was observed in the RDFs as the concentrations of graphene and hexagonal boron nitride nanoparticles increased. The high number of nanoparticles led to larger distances between the centers of mass of the nanoparticles, indicating a deterioration of long-range order. When the RDFs of graphene oxide nanoparticles were analyzed, a single prominent peak was observed when *N_nf_* was either 24 or 48. This finding confirms the presence of better dispersion of graphene oxide nanoparticles in the PI nanocomposites. At the same time, the aggregates composed of graphene and hexagonal boron nitride nanoparticles possessed a more elongated spatial configuration.

**Figure 4:**
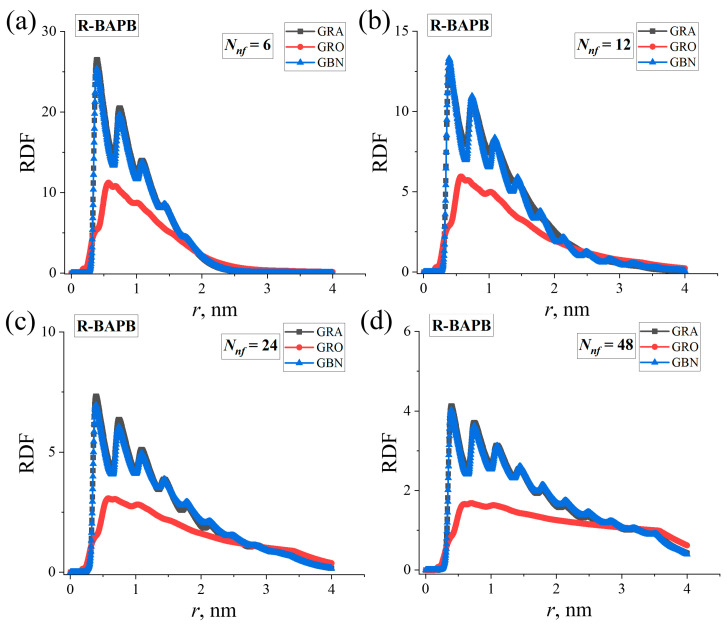
Intermolecular radial distribution functions (RDFs) of the atoms of graphene (‘GRA’), graphene oxide (‘GRO’), and hexagonal boron nitride (‘GBN’) molecules in PI nanocomposite systems based on R-BAPB for various numbers of *N_nf_*: (**a**) 6, (**b**) 12, (**c**) 24, and (**d**) 48 nanofiller molecules.

Intermolecular RDFs were also calculated for the atoms of the three types of nanofillers at *T* = 800 K. Figure 4 displays the intermolecular RDFs of the atoms of nanoparticles in nanocomposites based on PI R-BAPB. Similar relationships were observed for nanocomposites based on PIs BPDA-P3 and ULTEM^TM^, as shown in Figures S7 and S8 in the Supplementary Materials.

The results demonstrated that the intermolecular RDFs of atoms of graphene and boron nitride nanoparticles exhibited multiple peaks. The positions of the peaks and heights of the RDFs between the intermolecular RDFs of the graphene and boron nitride nanoparticles exhibited slight differences. The first peak occurred at a distance of ~ 0.4 nm, and the height of the peaks diminished as the distance between the nanoparticles increased. The intermolecular RDFs of atoms of graphene oxide showed a distinct difference; they displayed a single peak with a height significantly lower than that observed for intermolecular RDFs of atoms of graphene and boron nitride nanoparticles. As the nanoparticle concentration increased, the interactions between the atoms of graphene and boron nitride molecules became more pronounced. This led to an increase in the number of peaks and a slight decrease in the distance between the peaks. In the case of graphene oxide, a single peak was observed in the intermolecular RDFs, indicating the absence of stable long-range order and better dispersion of graphene oxide nanoparticles in the polymer matrix. The presence of several peaks in the intermolecular RDFs of graphene and boron nitride nanoparticles may indicate the formation of stable aggregates of these molecules that extend over a large distance, comparable to the size of the simulation box.

**Figure 5:**
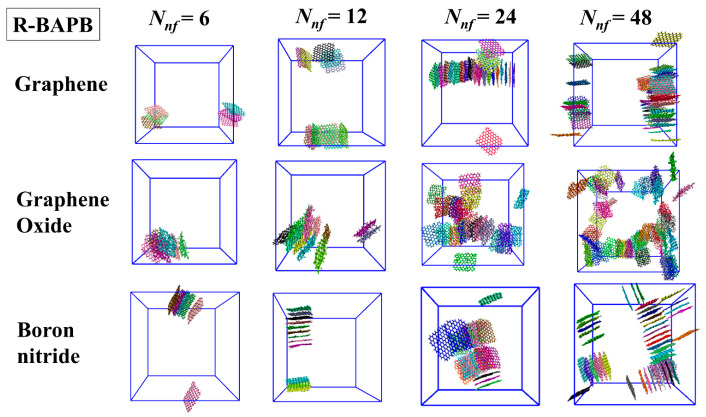
Representative snapshots of nanofiller molecules in R-BAPB polyimide nanocomposites based on graphene, graphene oxide, and hexagonal boron nitride with various numbers of nanofiller molecules at *T* = 800 K. For clarity, only nanofiller molecules are depicted, polyimide chains are omitted from the representation. Various nanofiller molecules are highlighted in different colors.

In order to substantiate this hypothesis, the immediate structures of the polyimide nanocomposites were examined at *T* = 800 K. Figure 5 shows the instantaneous configurations of graphene, graphene oxide, and boron nitride nanoparticles with various numbers of *N_nf_* molecules only in the R-BAPB nanocomposites. Similar instantaneous configurations of these nanoparticles in BPDA-P3 and ULTEM^TM^ nanocomposites are shown in Figures S9 and S10 in the Supplementary Materials.

Analysis of the obtained data shows that graphene and boron nitride nanoparticles tend to form elongated aggregates, apparently leading to the deterioration of their dispersion in the polymer binder. Graphene oxide molecules exhibited less prominent columnar structures of nanoparticles due to the presence of hydroxyl and epoxy groups on their surfaces. These nanoparticles adhered primarily to each other through their edges. Increasing the concentration of nanoparticles in the case of graphene and boron nitride led to an increase in the size of stacked aggregates formed from these nanoparticles, while graphene oxide nanoparticles formed less ordered aggregates.

**Figure 6:**
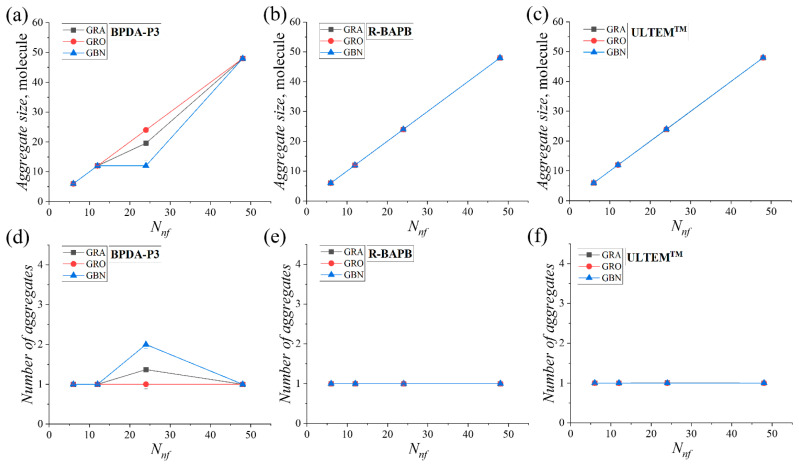
Average number of nanofiller molecules per aggregate (**a**–**c**) and average number of aggregates (**d**–**f**) as a function of the number of graphene, graphene oxide, and hexagonal boron nitride molecules in the polyimide nanocomposites at *T* = 800 K.

The results of the quantitative analysis of the average aggregate size and number of aggregates in the considered systems confirmed the increase in the size of the nanoparticle aggregates, Figure 6. 

It was found that the sizes of the aggregates exhibited a nearly linear relationship with the number of nanoparticles. Also, an increase in the nanofiller concentration of nearly all systems resulted in the formation of a single large aggregate. With the exception of the BPDA-P3-based nanocomposites with graphene and boron nitride nanoparticles for *N_nf_* = 24, a decrease in aggregate size (Figure 6a) and an increase in the average number of aggregates (Figure 6d) were observed. The results indicate that it is more favorable for all three types of nanoparticles to combine and form a single large aggregate surrounded by a polyimide binder.

Recent studies [73,74,75] have revealed the presence of stacked aggregates similar to those found in graphene and boron nitride nanoparticles within the modified nanoparticles of asphaltene molecules after the elimination of aliphatic side chains.

### 3.2. Thermophysical Properties

**Figure 7:**
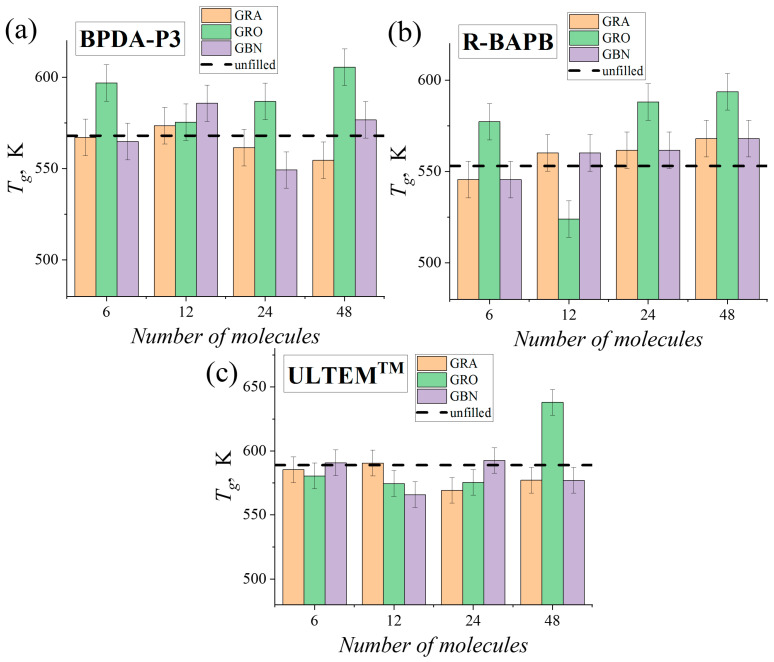
The glass transition temperature *T_g_* of the PI nanocomposites based on PIs (**a**) BPDA-P3, (**b**) R-BAPB, and (**c**) ULTEM^TM^ at various number of graphene (‘GRA’), graphene oxide (‘GRO’), and hexagonal boron nitride (‘GBN’) molecules. The horizontal dashed lines indicate the values of *T_g_* of the unfilled PIs [49].

In order to examine the impact of the nanofiller mass fraction on the thermophysical characteristics of the studied PI nanocomposites, the samples were cooled from 800 to 290 K. Figure S11 in the Supplementary Materials illustrates the density–temperature relationship *ρ*(*T*) during the cooling process for polyimide nanocomposites with various nanofiller mass fractions. The *ρ*(*T*) dependence exhibited two distinct linear regions at both high and low temperatures. The values of *T_g_* were determined for nanocomposites with various nanofiller mass fractions by analyzing the density–temperature dependence. The results are shown in Figure 7.

Analysis of the data presented in Figure 7 demonstrates that increasing the mass fraction of the nanofiller in polymer nanocomposites leads to a nonmonotonic behavior of value of *T_g_*. The addition of three types of nanoparticles to semicrystalline PIs BPDA-P3 and R-BAPB slight increased the values of *T_g_* of their nanocomposites only at high numbers of nanoparticle molecules (*N_nf_* = 24 and 48, respectively). Also, the increase in mass fraction of nanoparticles in polymer nanocomposite results in a reduction of nanoparticle mobility, Figures S2–S4 in the Supplementary Materials.

Among the studied nanoparticles, the graphene oxide exhibited the strongest increase in value of *T_g_* of polyimide nanocomposites relative to the unfilled sample. However, for nanocomposites based on amorphous PI ULTEM^TM^, the values of *T_g_* of all nanocomposites (with the exception of graphene oxide with *N_nf_* = 48 when a strong increase in value of *T_g_* was observed) was slightly lower than or comparable to that of the unfilled sample of this PI. Thus, the incorporation of graphene oxide nanoparticles into the polymer binder of PIs resulted in a more substantial enhancement in value of *T_g_*. This effect can be attributed to the improved dispersion of the graphene oxide nanoparticles. Mitra et al. [82] showed that a decrease in the mobility of the polymer chain could lead to an increase in the value of *T_g_* of nanocomposites based on poly(methyl methacrylate) and fullerene. In current study, the addition of graphene oxide nanoparticles to polymer nanocomposites resulted in a reduction in the mobility of polymer chains when the temperature exceeded value of *T_g_*, as shown in Figures S2–S4 in the Supplementary Materials. 

Establishing a correlation between the alteration in glass transition temperature due to the incorporation of nanoparticles and the variation in thermal conductivity coefficient of composite materials is essential for the fabrication of novel thermal interface materials. Therefore, the influence of nanofiller mass fraction on the variation in thermal conductivity in polyimide nanocomposites was investigated.

**Figure 8:**
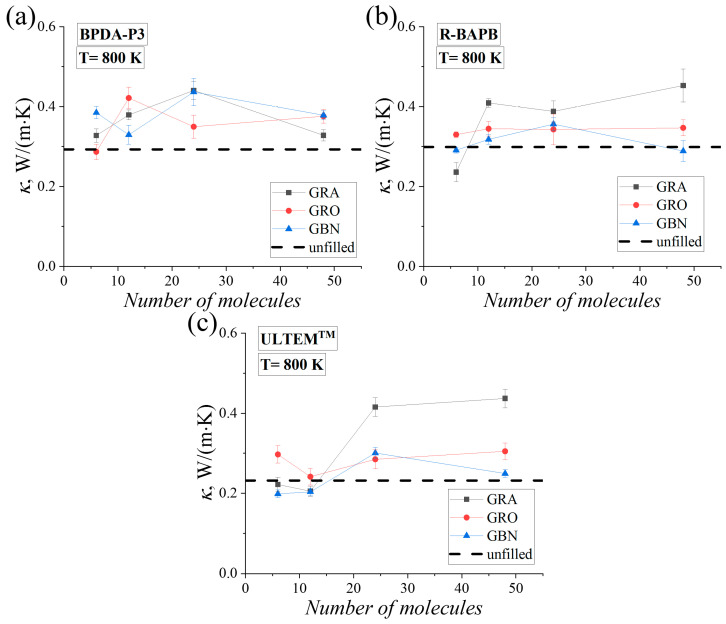
The thermal conductivity coefficients *κ* of the (**a**) BPDA-P3, (**b**) R-BAPB, and (**c**) ULTEM^TM^ nanocomposites as a function of the number of molecules (*N_nf_*) of graphene (‘GRA’), graphene oxide (‘GRO’), and hexagonal boron nitride (‘GBN’) in the samples at *T* = 800 K. Horizontal dashed lines show the values of *κ* of the unfilled PI samples [49].

Based on previous studies [73,74], primary focus was on the thermal conductivity coefficient of systems at temperatures above and below their transition point from the liquid to the solid states. The thermal conductivity coefficient *κ* was calculated using the instantaneous configuration of PI nanocomposites generated at *T* = 800 and 290 K. Figure 8 displays the values of *κ* of the PI nanocomposites with various numbers of *N_nf_* nanofiller molecules at a temperature of 800 K. The values of *κ* of polymer nanocomposites at *T* = 290 K are shown in Figure S12 in the Supplementary Materials.

The data analysis reveals that as the mass fraction of the nanofiller increases, there is a corresponding increase in the value of *κ* of the studied PI nanocomposites. The increase in the value of *κ*, as well as the value of *T_g_*, exhibits a nonmonotonic character with an increasing *N_nf_*. For the PI BPDA-P3, an increase in the mass fraction of the three nanofillers led to a rise in the value of *κ* for all BPDA-P3-based nanocomposites. A strong increase in the thermal conductivity coefficient began to occur compared to the unfilled samples of all three PI nanocomposites, similar to *T_g_* when the number of nanoparticles increased (*N_nf_* = 24 and 48). With the exception of the nanocomposites based on the PIs R-BAPB and ULTEM^TM^, when *N_nf_* = 48, a slight deterioration in the values of *κ* was observed. For nanocomposites based on PIs R-BAPB and ULTEM^TM^, the increase in the mass fraction of graphene led to value of *κ* was greater than that for nanocomposites based on hexagonal boron nitride and graphene oxide nanoparticles. An analogous increase in the value of *κ* of the polymer nanocomposite was observed with increasing mass fractions of graphene [28], graphene oxide [83], and hexagonal boron nitride [37] nanofiller for other polymers. 

It is important to note that the value of *κ* in the glassy state may vary depending on the cooling rate [56]. Since the polymer chains adopt different conformations to the glassy state during the cooling process, the effect of the mass fraction change of the nanofiller on the thermal conductivity of polyimide nanocomposites in the glassy state can vary significantly. This is primarily due to the significant structural differences of polymers near the surface of the nanoparticles, which consequently results in differences in the thermal conductivities of polymer nanocomposites. After cooling BPDA-P3-based nanocomposites, their thermal conductivity coefficient slightly increased with increasing nanofiller mass fraction, resulting in an enhancement in the thermal conductivity at *T* = 290 K. The incorporation of nanoparticles tends to improve the overall thermal conductivity of PI nanocomposites, although a slight decrease in value of *κ* was observed at the maximum concentration with graphene nanoparticles, Figure S12a in the Supplementary Materials. A similar trend was observed for R-BAPB-based nanocomposites, with increasing graphene and boron nitride nanoparticle mass fractions leading to increased thermal conductivity, although a slight decrease was observed in value of *κ* at the maximum graphene oxide nanoparticle concentration, Figure S12b in the Supplementary Materials. For the ULTEM-based nanocomposites, increasing the graphene and boron nitride nanoparticle concentrations also led to an increase in the value of *κ*, whereas the addition of graphene oxide nanoparticles led to a deterioration in thermal conductivity compared to unfilled ULTEM sample, Figure S12c in the Supplementary Materials. In general, for PI nanocomposites at *T* = 290 K, it was observed that the addition of hexagonal nanoparticles (primarily graphene and boron nitride) resulted in an enhancement in the thermal conductivity of the PI nanocomposites, Figure S12 in the Supplementary Materials.

The dissimilarity in the thermal conductive properties of the aggregates comprising graphene and hexagonal boron nitride might be attributed to the difference in their chemical structure as well as the shape of the aggregates of these nanoparticles. The more compact packing of hexagonal boron nitride nanoparticles (Figure 3 and Figure 4) in the aggregates may hinder the transmission of phonons owing to the vibration restriction of atoms in the plane of these nanoparticles compared to those of graphene nanoparticles, which could explain the observed difference in thermal conductivity.

The results of the simulations can be extrapolated to experimental systems even when the quantity of additional nanoparticles in the polymer sample significantly exceeds that in the modeled systems. The developed model demonstrates a general trend in the alteration of the thermal conductivity properties of polymer nanocomposite materials when the incorporation of graphene, graphene oxide and boron nitride nanoparticles was performed. This qualitatively aligns with the experimentally observed increase in the thermal conductivity. Future plans include the development and investigation of performance properties of nanocomposite models using thermoplastic polyimides reinforced with mixtures of hexagonal nanoparticles.

## 4. Conclusions

This study investigated the thermophysical, structural, and dynamic properties of semicrystalline R-BAPB and BPDA-P3 as well as amorphous ULTEM^TM^ polyimide nanocomposites incorporating various nanofillers using all-atom molecular dynamics simulations. The three most commonly used nanofiller materials—graphene, graphene oxide, and hexagonal boron nitride nanoparticles—were examined. The results showed that the formation of stacked aggregates from graphene and hexagonal boron nitride nanoparticles maintained their order over long distances. Graphene oxide nanoparticles showed less ordered aggregates but a more effective distribution within the polymer nanocomposites. The mass fraction of the nanofillers had a slight influence on the number of aggregates.

The translational mobility of the nanofillers in the polymer nanocomposites decreased with an increase in the nanofiller mass fraction. As a result of the better interaction between the graphene oxide nanoparticles and the polyimide chains, an increase in the mass fraction of these nanoparticles led to a decrease in the translational mobility of the polymer chains of the studied polyimides. This decrease in polymer chain mobility was correlated with an increase in the glass transition temperature of the polyimide nanocomposites. An increase in the size of the nanoparticle aggregates resulted in an increase in the thermal conductivity coefficient at temperatures both above and below the glass transition temperature. Incorporation of graphene nanoparticles in polyimide nanocomposites yielded a more substantial enhancement of their thermal conductivity compared to the addition of hexagonal boron nitride or graphene oxide nanoparticles.

## Figures and Tables

**Table 1:** Characterization of the systems considered.

System	Number of Polymer Chains	Number of Nanofiller Molecules, *N_nf_*	Type of Nanofiller Molecules	Mass/Volume Fraction of Nanofiller C_np_(%) for Polyimides R-BAPB and BPDA-P3	Mass/Volume Fraction of Nanofiller C_np_(%) for Polyimide ULTEM^TM^
PI-GRA-6	27	6	Graphene	4.9/4.8	6.3/4.6
PI-GRA-12	27	12	Graphene	9.3/9.1	11.9/8.8
PI-GRA-24	27	24	Graphene	16.9/16.7	21.2/16.2
PI-GRA-48	27	48	Graphene	29/28.6	35/27.9
PI-GRO-6	27	6	Graphene oxide	6.4/8.1	8.2/7.8
PI-GRO-12	27	12	Graphene oxide	12/15.2	15.2/14.7
PI-GRO-24	27	24	Graphene oxide	21.4/27	26.4/26.3
PI-GRO-48	27	48	Graphene oxide	35.2/43.9	41.8/43
PI-GBN-6	27	6	Boron nitride	4.9/7	6.3/6.7
PI-GBN-12	27	12	Boron nitride	9.3/13.2	11.9/12.8
PI-GBN-24	27	24	Boron nitride	16.9/23.9	21.2/23.2
PI-GBN-48	27	48	Boron nitride	29/39.7	35/38.7

## Data Availability

The data presented in this study are available upon request from the corresponding author. The data are not publicly available due to the large size of simulation trajectories.

## Supplementary Materials

The following supporting information can be downloaded at: https://www.mdpi.com/article/10.3390/polym16233231/s1, Figure S1: Radius of gyration of polymer chains of different polyimide nanocomposites based on (a–c) BPDA-P3, (d–f) R-BAPB, and (g–i) ULTEM^TM^ and (a,d,g) graphene, (b,e,h) graphene oxide, and (c,d,i) hexagonal boron nitride for various numbers of nanofiller molecules (*N_nf_*) as a function of time. Figure S2: Mean squared displacement of the center mass of the nanofiller (‘NCM’) molecules (a–c) (graphene (‘GRA’), graphene oxide (‘GRO’), and hexagonal boron nitride (‘GBN’)) and (d–f) polyimide chain in the BPDA-P3 polyimide nanocomposites with various numbers of nanofiller molecules (*N_nf_*). Figure S3: Mean squared displacement of center of mass of nanofiller (‘NCM’) (a–c) molecules (graphene (‘GRA’), graphene oxide (‘GRO’), and hexagonal boron nitride (‘GBN’)) and (d-f) R-BAPB polyimide chain (‘PCM’) with various numbers of nanofiller molecules (*N_nf_*). Figure S4: Mean squared displacement of the center of mass of nanofiller (a–c) molecules (graphene (‘GRA’), graphene oxide (‘GRO’), and hexagonal boron nitride (‘GBN’)) and (d–f) polyimide chain (‘PCM’) in the ULTEM^TM^ polyimide nanocomposites with various numbers of nanofiller molecules (*N_nf_*). Figure S5: Radial distribution functions (RDF)s for the centers of mass of polycyclic cores of graphene molecules (‘GRA’), graphene oxide (‘GRO’), and hexagonal boron nitride (‘GBN’) in polyimide nanocomposite systems based on BPDA-P3 for various numbers of nanofiller molecules (*N_nf_*): (a) 6, (b) 12, (c) 24, and (d) 48. Figure S6: Radial distribution functions (RDF)s for the centers of mass of polycyclic cores of graphene molecules (‘GRA’), graphene oxide (‘GRO’), and hexagonal boron nitride (‘GBN’) in polyimide nanocomposite systems based on ULTEM^TM^ for various numbers of nanofiller molecules (*N_nf_*): (a) 6, (b) 12, (c) 24, and (d) 48. Figure S7: The intermolecular radial distribution functions (RDF)s of graphene (‘GRA’), graphene oxide (‘GRO’), and hexagonal boron nitride (‘GBN’) molecules in BPDA-P3-based polyimide nanocomposite systems for various numbers of nanofiller molecules (*N_nf_*): (a) 6, (b) 12, (c) 24, and (d) 48. Figure S8: The intermolecular radial distribution functions (RDF) of graphene (‘GRA’), graphene oxide (‘GRO’), and hexagonal boron nitride (‘GBN’) molecules in ULTEM^TM^-based polyimide nanocomposite systems for various numbers of nanofiller molecules (*N_nf_*): (a) 6, (b) 12, (c) 24, and (d) 48. Figure S9: Representative snapshots of nanofiller molecules in BPDA-P3 polyimide nanocomposites based on graphene, graphene oxides, and boron nitride for various numbers of nanofiller molecules (*N_nf_*). Figure S10: Representative snapshots of nanofiller molecules in ULTEM^TM^ polyimide nanocomposites based on graphene, graphene oxides, and boron nitride for various numbers of nanofiller molecules (*N_nf_*). Figure S11: The mass density of (a–c) BPDA-P3, (d–f) R-BAPB, and (g–i) ULTEM^TM^ polyimide as a function of temperature for nanocomposite samples with various nanofillers (graphene (‘GRA’), graphene oxide (‘GRA’), and hexagonal boron nitride (‘GBN’)) for different numbers of nanofiller molecules (*N_nf_*). Figure S12: Thermal conductivity coefficients *κ* of the (a) BPDA-P3, (b) R-BAPB, and (c) ULTEM^TM^ nanocomposites as a function of the number of nanofiller molecules (*N_nf_*) in the sample at *T* = 290 K.
