# Supplementary material for: Enhanced Thermal Conductivity of Thermoplastic Polyimide Nanocomposites: Effect of Using Hexagonal Nanoparticles
Source: Polymers (Basel). 2024 Nov 21;16(23):3231. doi: 10.3390/polym16233231 (PMC11644118; doi:10.3390/polym16233231)
Supplement: Supplementary file 1 [file polymers-16-03231-s001.zip › polymers-3312509-supplementary.pdf]

Supplementary Materials

for

Enhanced Thermal Conductivity of Thermoplastic Polyimide Nanocomposites: Effect of Using Hexagonal Nanoparticles

Victor M. Nazarychev*

Branch of Petersburg Nuclear Physics Institute named by B.P.Konstantinov of National Research Centre «Kurchatov Institute» – Institute of Macromolecular Compounds, Bolshoi, pr. 31 (V.O.) 199004 St. Petersburg, Russia.

*Corresponding author: Victor M. Nazarychev; e-mail: nazarychev@imc.macro.ru, tel: +7 (812) 3230216

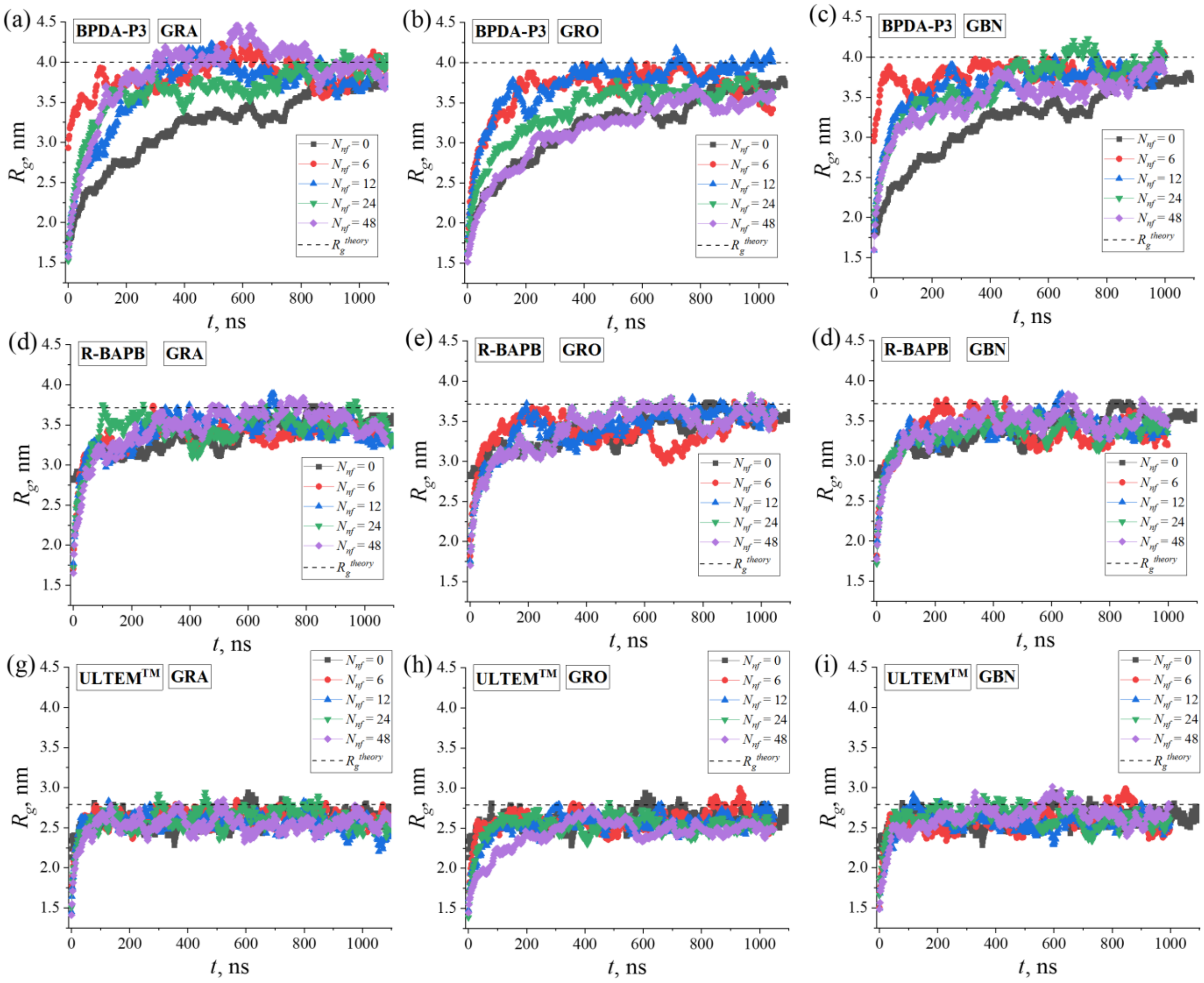

Figure S1. Radius of gyration of polymer chains of different polyimide nanocomposites based on (a-c) BPDA-P3, (d-f) R-BAPB, (g-i) ULTEM^{TM} and (a,d,g) graphene, (b,e,h) graphene oxide, and (c,d,i) hexagonal boron nitride for various numbers of nanofiller molecules N_{nf} as a function of time. The dashed black horizontal lines correspond to the radius of gyration predicted analytically for the free joint model [1–3].

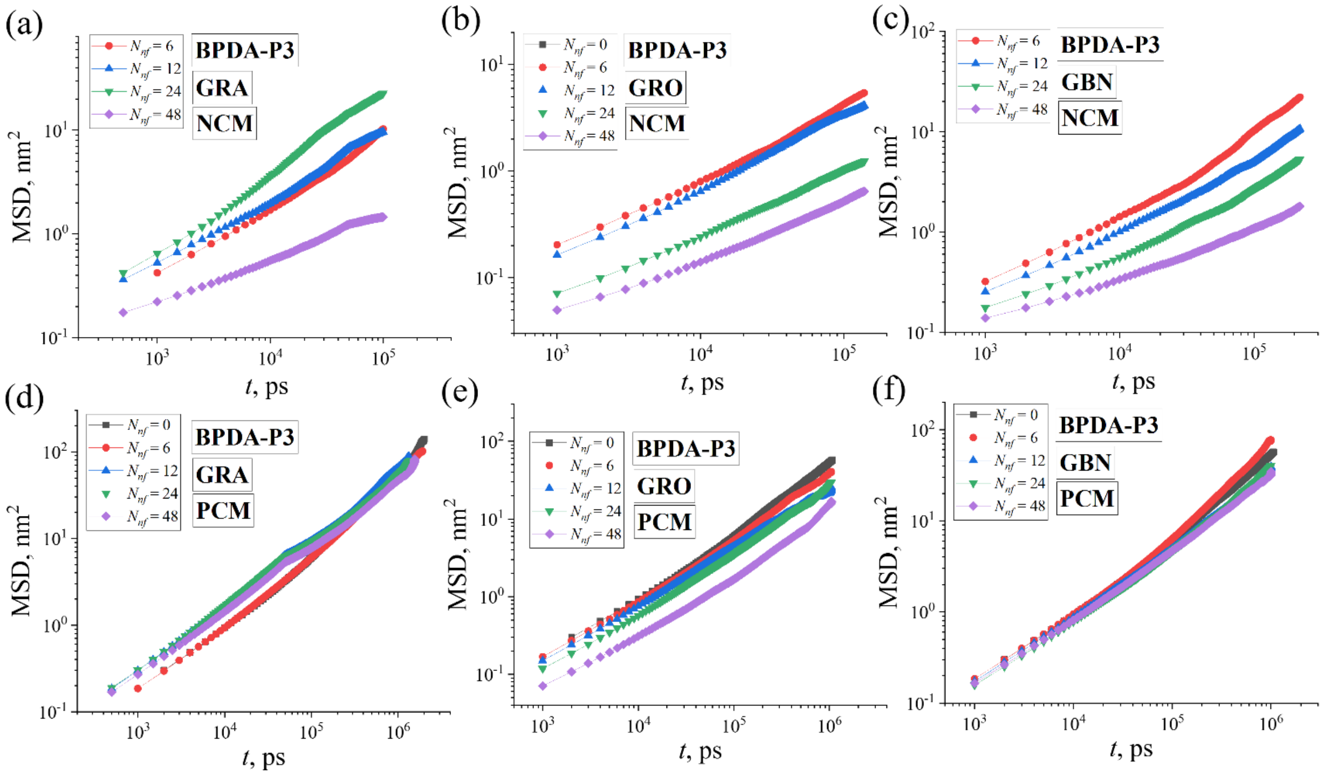

Figure S2. Mean squared displacement of the center mass of the nanofiller ('NCM') molecules (a-c) (graphene ('GRA'), graphene oxide ('GRO') and hexagonal boron nitride ('GBN')) and (d-f) polyimide chain ('PCM') in the BPDA-P3 polyimide nanocomposites with various numbers of nanofiller molecules N_{nf}. All calculations are performed at T = 800 K.

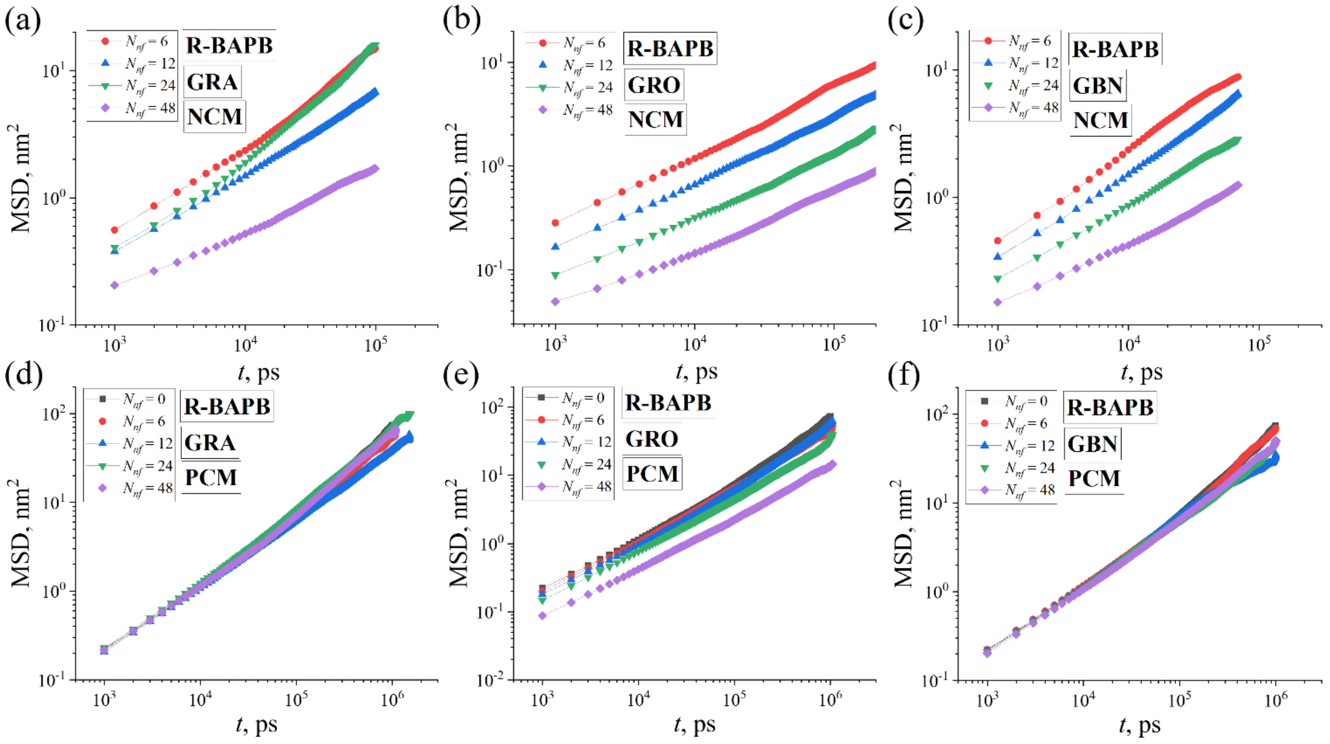

Figure S3. Mean squared displacement of center of mass of nanofiller ('NCM') molecules (a-c) (graphene ('GRA'), graphene oxide ('GRO'), and hexagonal boron

nitride ('GBN')) and (d-f) R-BAPB polyimide chain ('PCM') with various numbers of N_{nf} nanofiller molecules. All calculations were performed at T = 800 K.

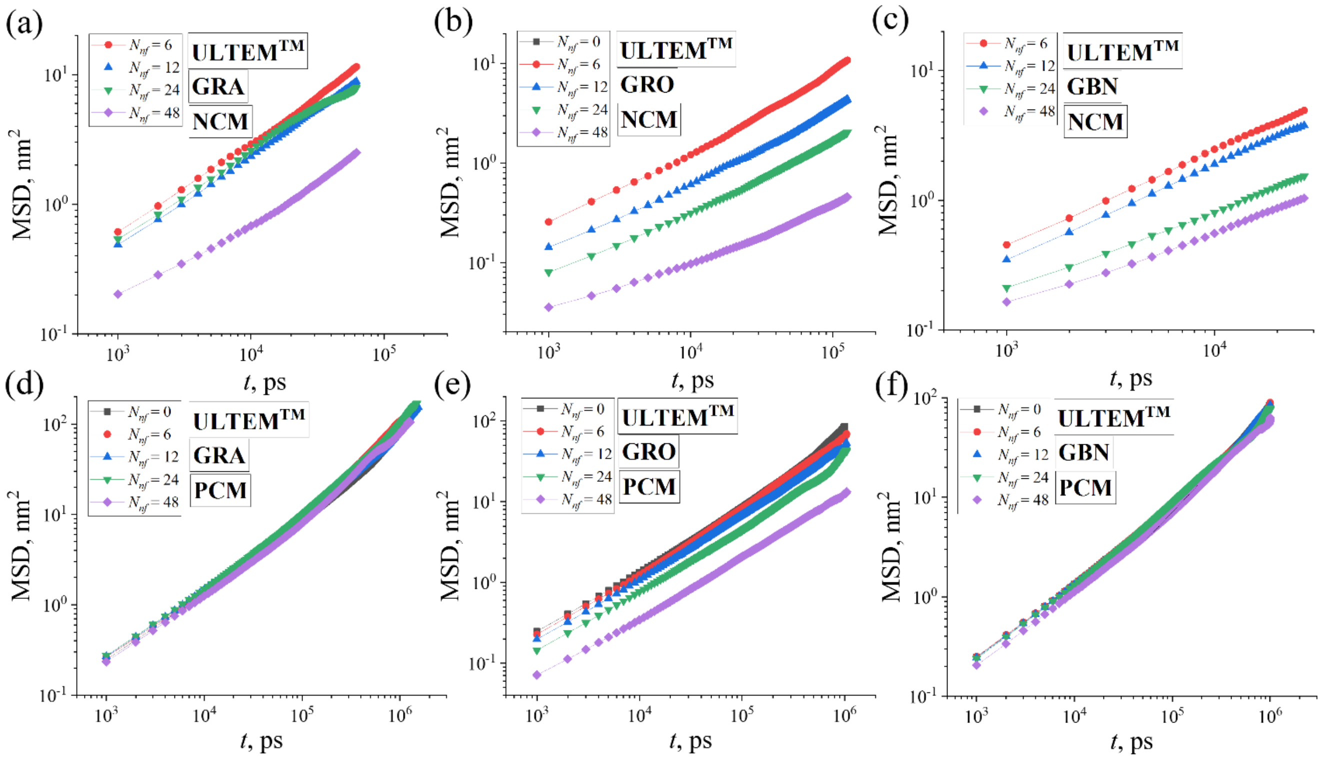

Figure S4. Mean squared displacement of the center of mass of nanofiller ('NCM') molecules (a-c) (graphene ('GRA'), graphene oxide ('GRO'), and hexagonal boron nitride ('GBN')) and (d-f) polyimide chain ('PCM') in the ULTEM™ polyimide nanocomposites with various numbers of N_{nf} nanofiller molecules. All calculations are performed at T = 800 K.

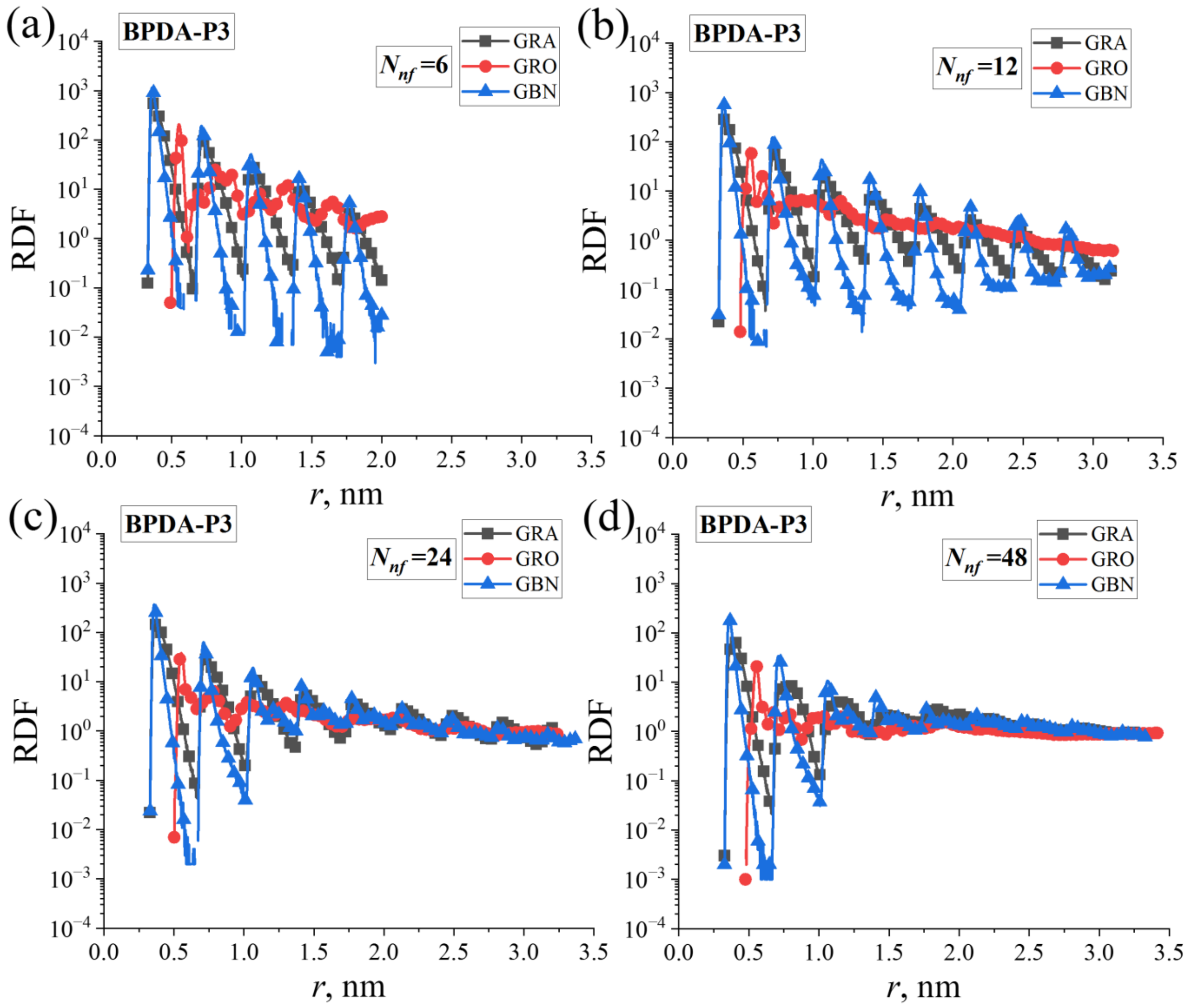

Figure S5. Radial distribution functions (RDF) for the centers of mass of polycyclic cores of graphene ('GRA'), graphene oxide ('GRO'), and hexagonal boron nitride ('GBN') molecules in polyimide nanocomposite systems based on BPDA-P3 at various numbers of N_{nf} (a) 6, (b) 12, (c) 24, and (d) 48 of nanofiller molecules.

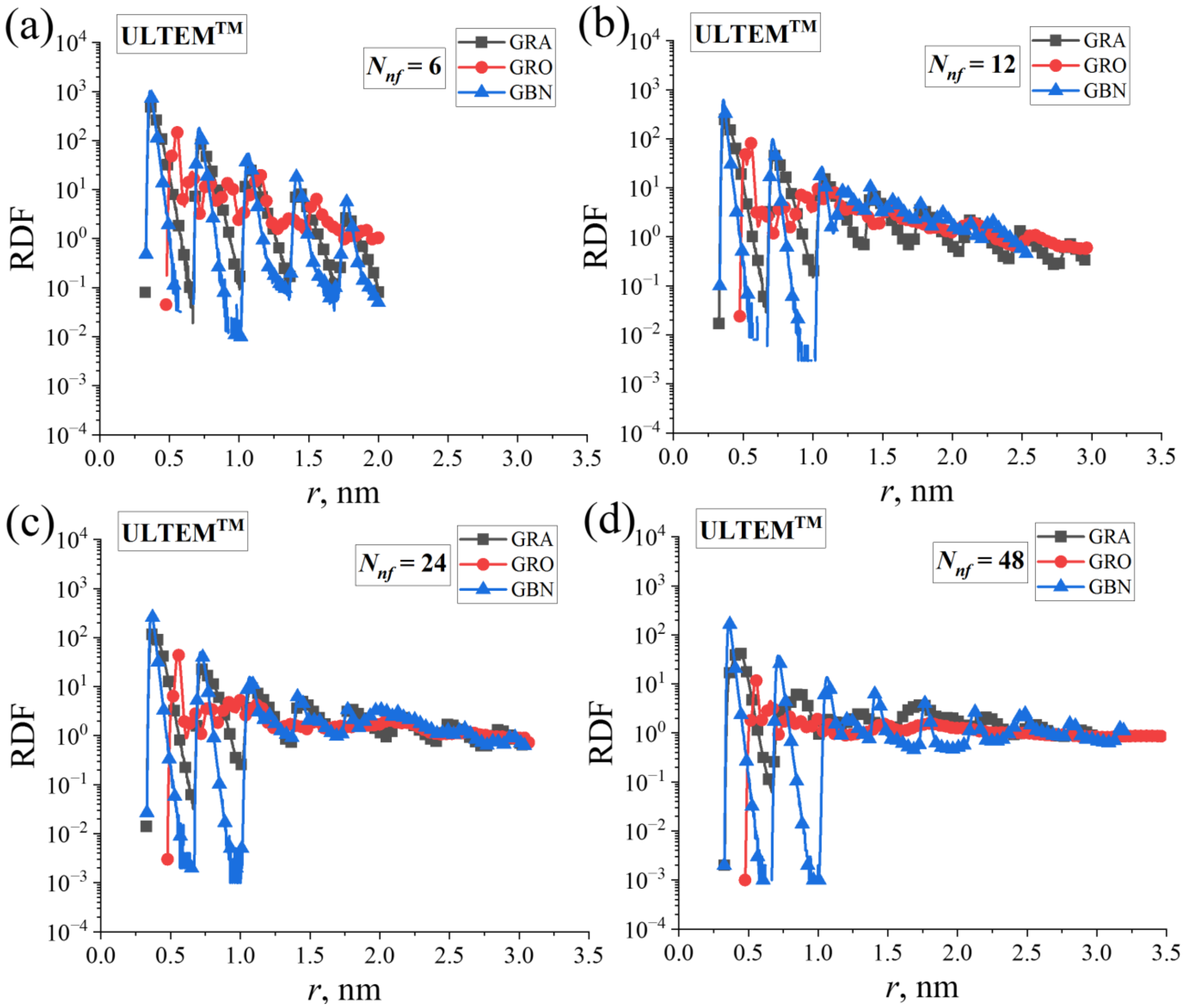

Figure S6. Radial distribution functions (RDF) for the centers of mass of polycyclic cores of graphene ('GRA'), graphene oxide (‘GRO’), and hexagonal boron nitride ('GBN') molecules in polyimide nanocomposite systems based on ULTEM™ at various numbers of N_{nf} (a) 6, (b) 12, (c) 24, and (d) 48 of nanofiller molecules.

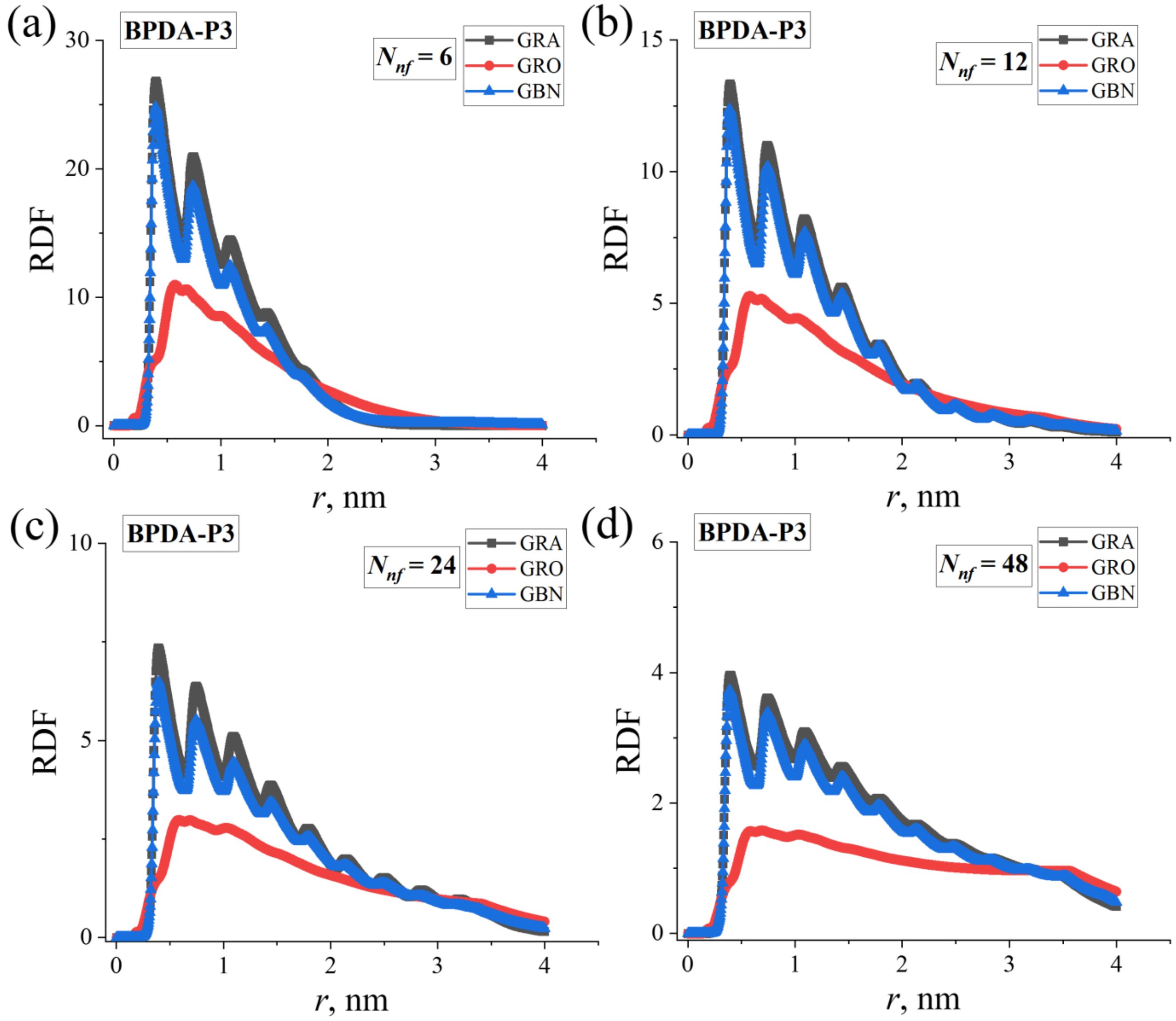

Figure S7. The intermolecular radial distribution functions (RDF) of graphene ('GRA'), graphene oxide ('GRO') and hexagonal boron nitride ('GBN') molecules in BPDA-P3-based polyimide nanocomposite systems at various numbers N_{nf} (a) 6, (b) 12, (c) 24, and (d) 48 of nanofiller molecules.

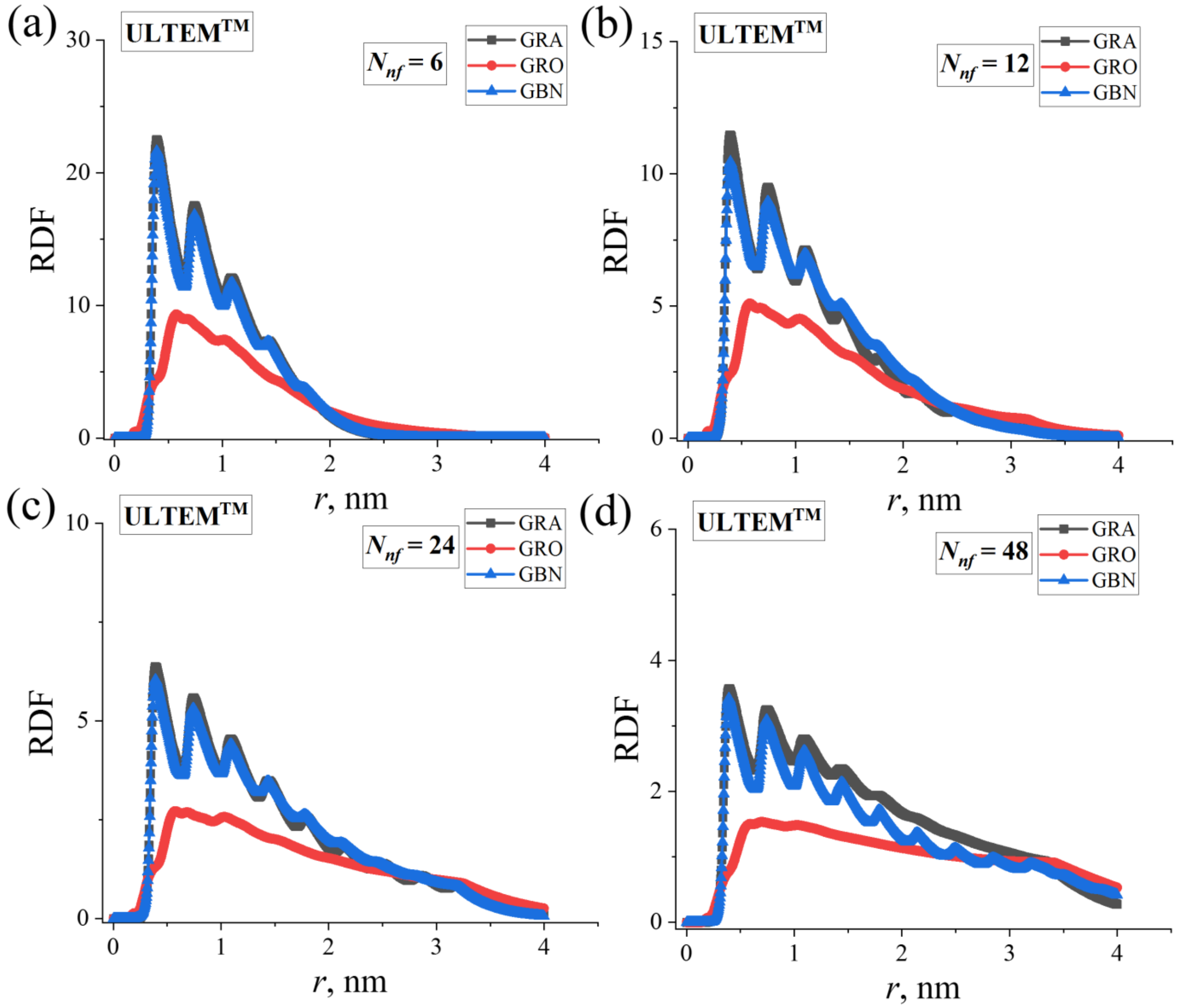

Figure S8. The intermolecular radial distribution functions (RDF) of graphene ('GRA'), graphene oxide ('GRO') and hexagonal boron nitride ('GBN') molecules in ULTEM^{TM}-based polyimide nanocomposite systems at various numbers N_{nf} (a) 6, (b) 12, (c) 24, and (d) 48 nanofiller molecules.

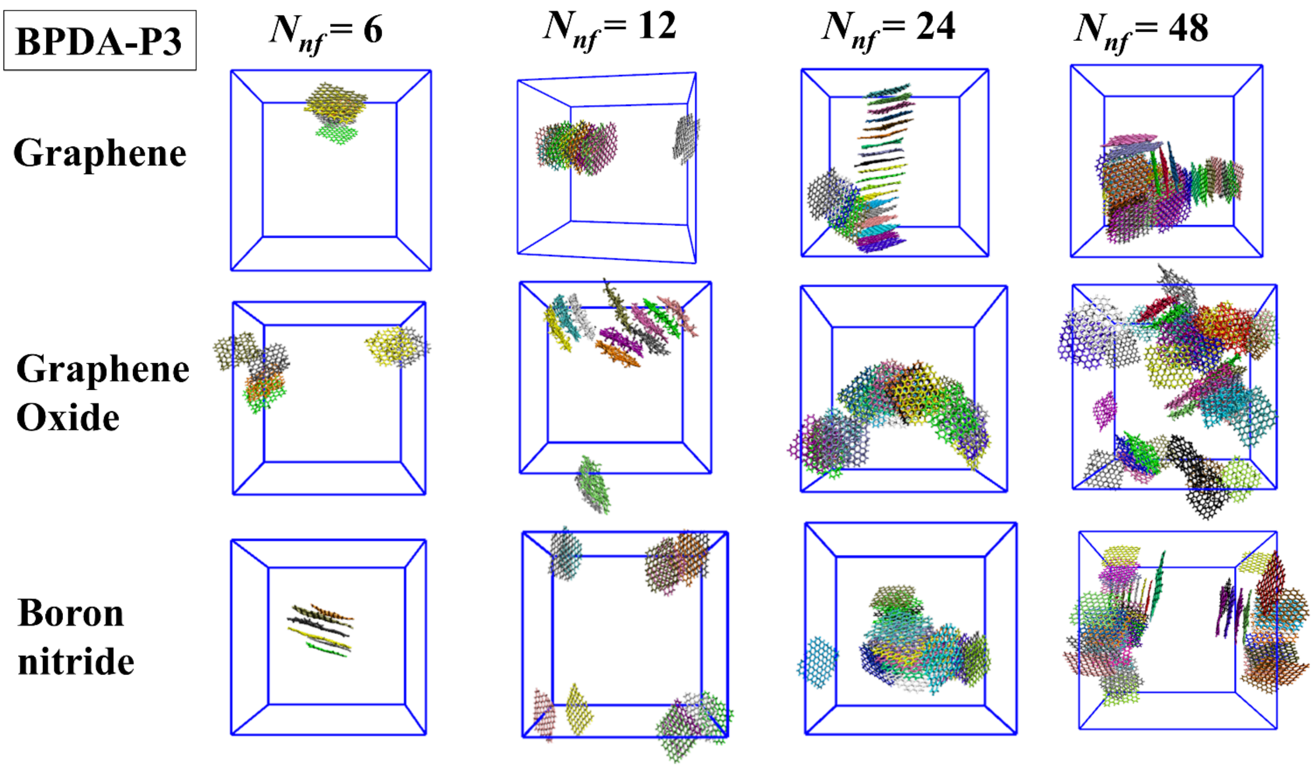

Figure S9. Representative snapshots of nanofiller molecules in BPDA-P3 polyimide nanocomposites based on graphene, graphene oxides, and boron nitride with various numbers *of* N_{nf} nanofiller molecules. For clarity, only nanofiller molecules are depicted, polyimide chains are omitted from the representation. Various nanofiller molecules are highlighted in different colors.

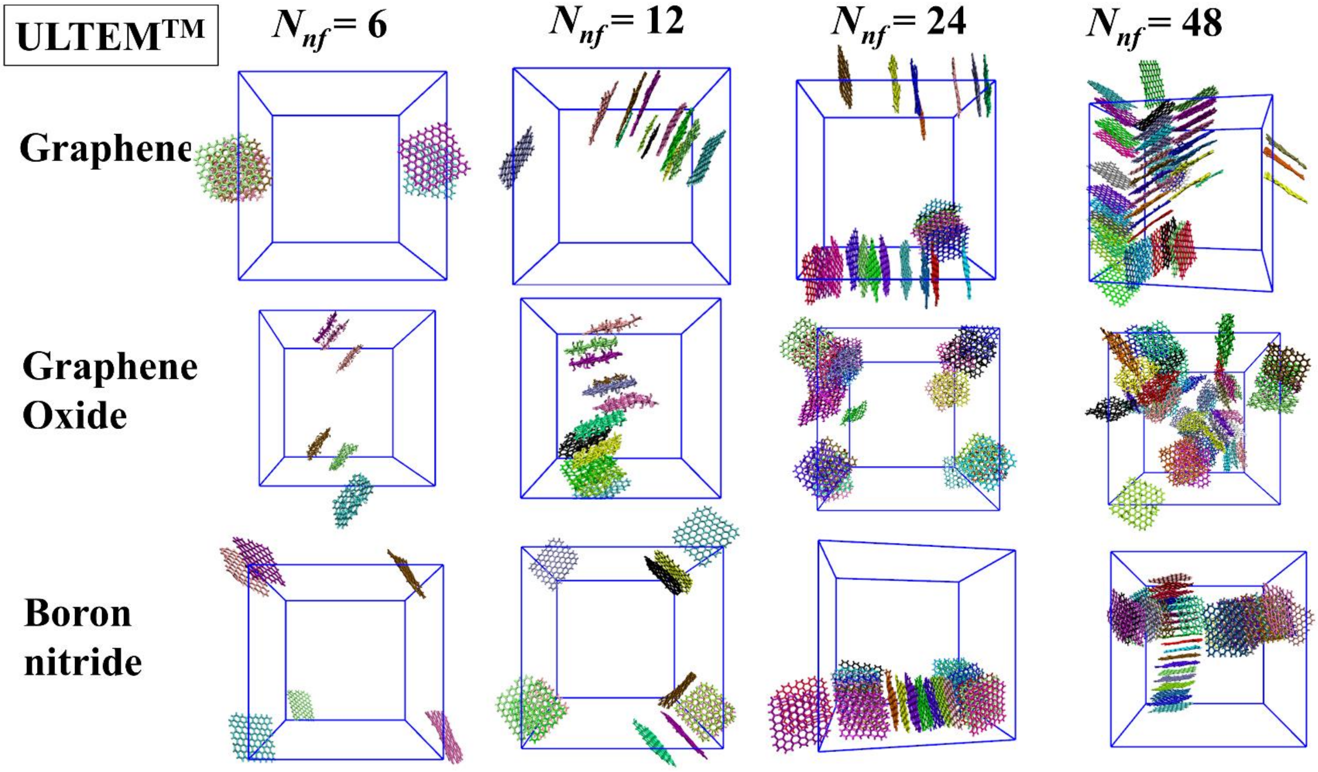

Figure S10. Representative snapshots of nanofiller molecules in ULTEM™ polyimide nanocomposites based on graphene, graphene oxides, and boron nitride with various numbers of N_{nf} nanofiller molecules. For clarity, only nanofiller molecules are depicted, polyimide chains are omitted from the representation. Various nanofiller molecules are highlighted in different colors.

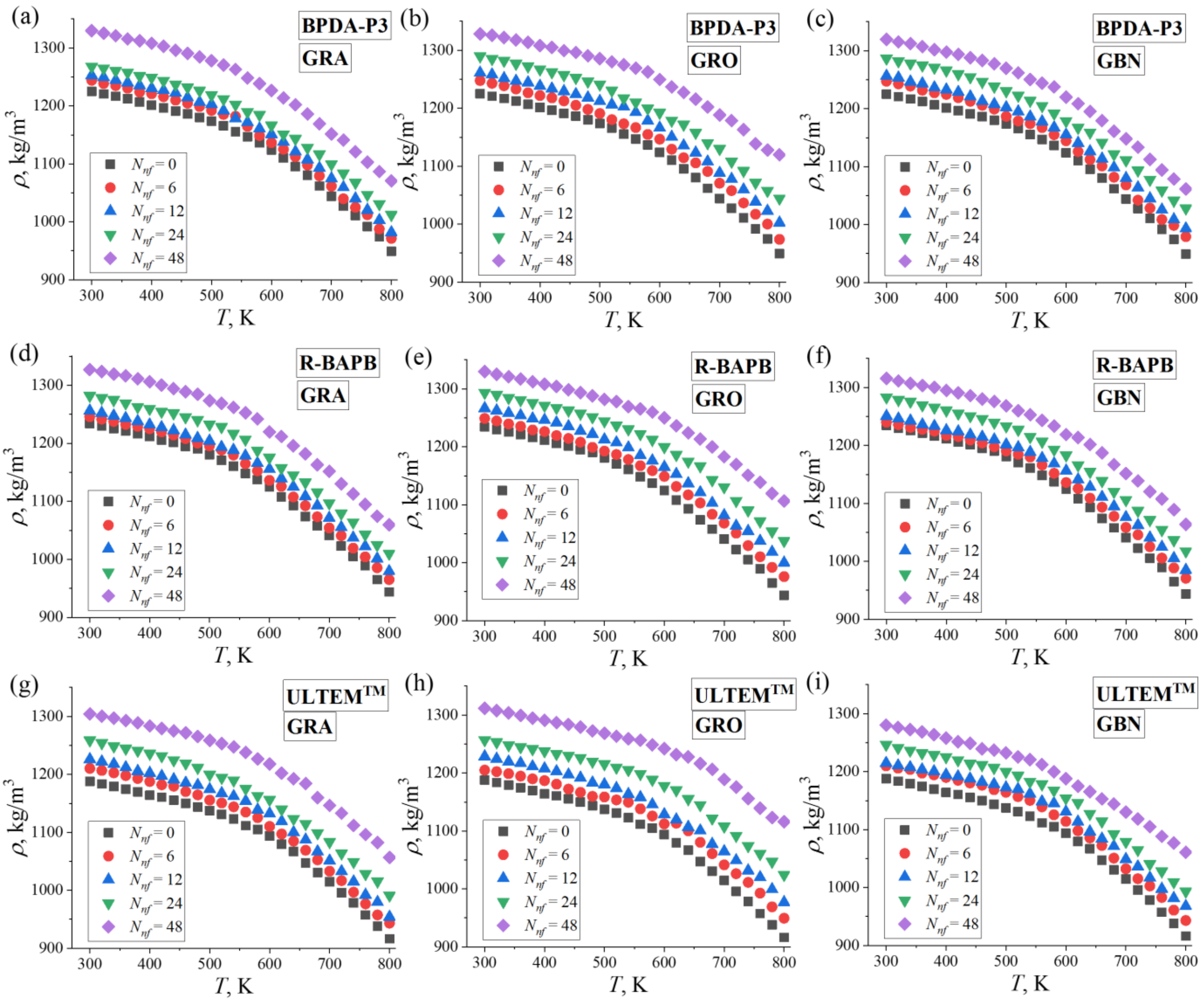

Figure S11. The mass density of (a-c) BPDA-P3, (d-f) R-BAPB, and (g-i) ULTEM™ polyimide as a function of temperature for nanocomposites samples with various nanofillers (graphene ('GRA'), graphene oxide ('GRA') and hexagonal boron nitride ('GBN')) at different numbers N_{nf} of nanofiller molecules.

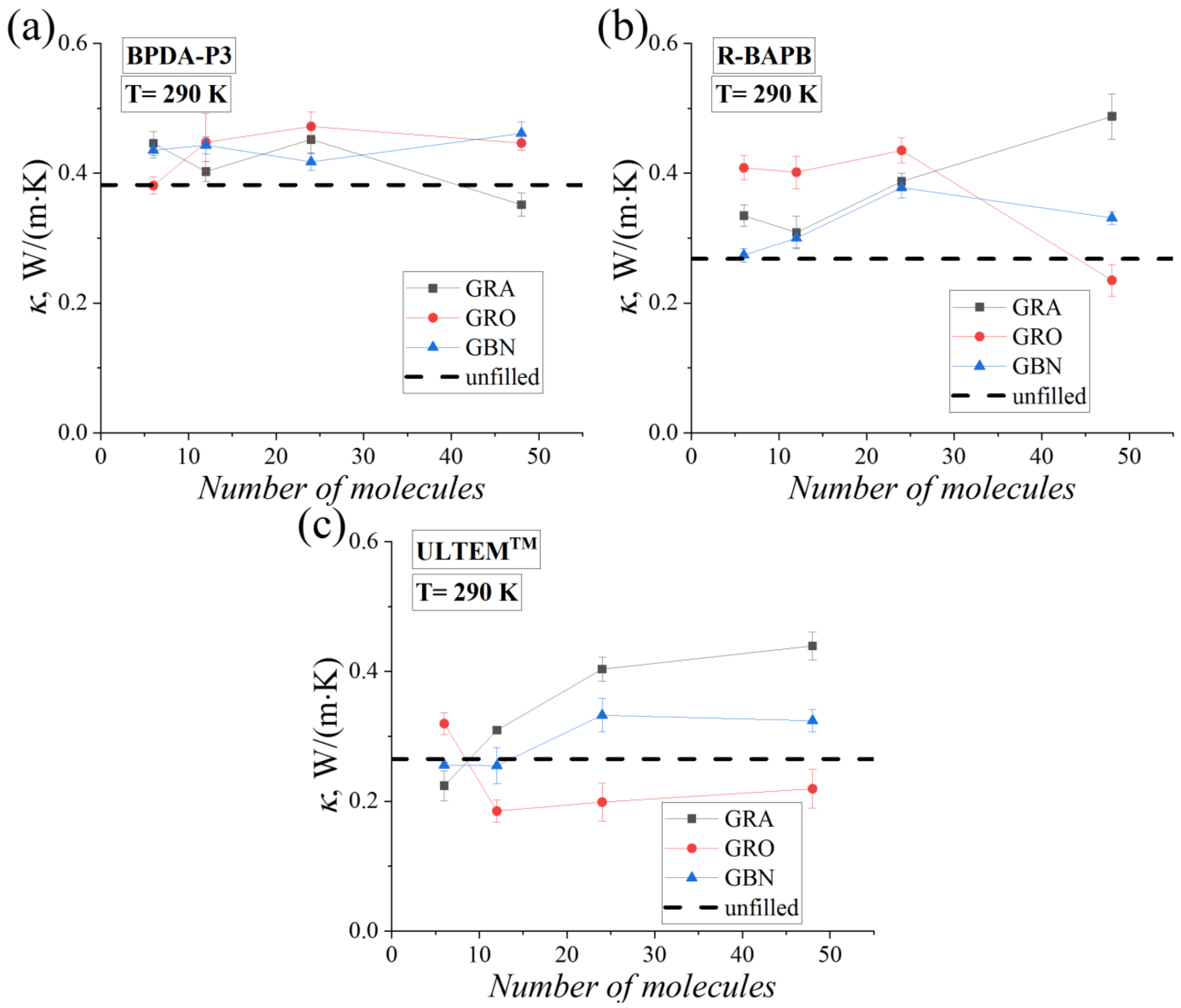

Figure S12. Thermal conductivity coefficients κ of the (a) BPDA-P3, (b) R-BAPB, and (c) ULTEM^{TM} nanocomposites samples as a function of the number of N_{nf} nanofiller molecules in the sample at T = 290 K. Horizontal black dashed lines show the κ values of the unfilled polyimide samples [4].

References.

1. Nazarychev, V.M.; Dobrovskiy, A.Y.; Larin, S. V.; Lyulin, A. V.; Lyulin, S. V. Simulating Local Mobility and Mechanical Properties of Thermostable Polyimides with Different Dianhydride Fragments. *J. Polym. Sci. Part B Polym. Phys.* **2018**, *56*, 375–382, doi:10.1002/polb.24550.
2. Larin, S. V.; Falkovich, S.G.; Nazarychev, V.M.; Gurtovenko, A.A.; Lyulin, A. V.; Lyulin, S. V. Molecular-Dynamics Simulation of Polyimide Matrix Pre-Crystallization near the Surface of a Single-Walled Carbon Nanotube. *RSC Adv.* **2014**, *4*, 830–844, doi:10.1039/C3RA45010D.
3. Falkovich, S.G.; Lyulin, S. V.; Nazarychev, V.M.; Larin, S. V.; Gurtovenko, A.A.; Lukasheva, N. V.; Lyulin, A. V. Influence of the Electrostatic Interactions on Thermophysical Properties of Polyimides: Molecular-Dynamics Simulations. *J. Polym. Sci. Part B Polym. Phys.* **2014**, *52*, 640–646, doi:10.1002/polb.23460.
4. Nazarychev, V.M.; Lyulin, S. V. The Effect of Mechanical Elongation on the Thermal Conductivity of Amorphous and Semicrystalline Thermoplastic Polyimides: Atomistic Simulations. *Polymers.* **2023**, *15*, 2926, doi:10.3390/polym15132926.
